# Supplementary material for: Low-Level Laser Treatment Induces the Blood-Brain Barrier Opening and the Brain Drainage System Activation: Delivery of Liposomes into Mouse Glioblastoma
Source: Pharmaceutics. 2023 Feb 8;15(2):567. doi: 10.3390/pharmaceutics15020567 (PMC9966329; doi:10.3390/pharmaceutics15020567)
Supplement: Supplementary file 1 [file pharmaceutics-15-00567-s001.zip › pharmaceutics-2167351-SI.pdf]

Supplementary information

Low-Level Laser Treatment Induces the Blood-Brain Barrier Opening and the Brain Drainage System Activation: Delivery of Liposomes into Mouse Glioblastoma

Oxana Semyachkina-Glushkovskaya [1,2,*], Denis Bragin [3,4], Olga Bragina [3], Sergey Socolovski [5], Alexander Shirokov [2,6], Ivan Fedosov [2], Vasily Ageev [2], Inna Blokhina [2], Alexander Dubrovsky [2], Valeria Telnova [2], Andrey Terskov [2], Alexander Khorovodov [2], Daria Elovenko [2], Arina Evsukova [2], Maria Zhoy [2], Ilana Agranovich [2], Elena Vodovozova [7], Anna Alekseeva [7], Jürgen Kurths [1,2,8] and Edik Rafailov [5]

1 Institute of Physics, Humboldt University, Newtonstrasse 15, 12489 Berlin, Germany
2 Department of Biology, Saratov State University, Astrakhanskaya 82, 410012 Saratov, Russia
3 Lovelace Biomedical Research Institute, Albuquerque, NM 87108, USA
4 Department of Neurology, University of New Mexico School of Medicine, Albuquerque, NM 87131, USA
5 Optoelectronics and Biomedical Photonics Group, Aston Institute of Photonic Technologies, Aston University, Birmingham B4 7ET, UK
6 Institute of Biochemistry and Physiology of Plants and Microorganisms, Russian Academy of Sciences, Prospekt Entuziastov 13, 410049 Saratov, Russia
7 Shemyakin-Ovchinnikov Institute of Bioorganic Chemistry, Russian Academy of Sciences, Miklukho-Maklaya 16/10, 117997 Moscow, Russia
8 Potsdam Institute for Climate Impact Research, Department of Complexity Science, Telegrafenberg A31, 14473 Potsdam, Germany
* Correspondence: glushkovskaya@mail.ru; Tel.: +7-8452-519220

Table S1. The 1268 nm-induced increase in the BBB permeability to the EBAC in healthy mice

**Laser 1268 nm intensities, mW**	**Content of EBAC in the brain (µg/g tissue)**		
	**1 h after 1268 nm irradiation**	**4 h after 1268 nm irradiation**	**24 h after 1268 nm irradiation**
No irradiation (the control group)	0.11±0.03 (0)	0.12±0.01	0.13±0.03
10	0.30±0.07 * (30)	0.14±0.02	0.11±0.01
20	0.48±0.01 ** (40)	0.11±0.04	0.13±0.01
30	0.46±0.04 ** (30)	0.12±0.01	0.14±0.02
40	0.48±0.03 ** (50)	0.10±0.07	0.11±0.02
50	0.58±0.01 *** (60)	0.12±0.04	0.11±0.03
60	0.6±0.01 *** (60)	0.15±0.08	0.12±0.05
**70**	**1.17**±0.09 *** (100)	0.11±0.01	0.14±0.04
**80**	**0.73**±0.07 *** (100)	0.16±0.06	0.12±0.08
**90**	**0.75**±0.03 *** (100)	0.13±0.04	0.11±0.03

*p>0.05; ** p>0.01; *** p>0.001 vs. the control group (no laser irradiation), n=10 in each group, the Mann–Whitney–Wilcoxon test; (0-100) – the number of mice with BBBO expressed as % of total number of mice, the selected area by green color illustrates 100% of mice with BBBO after laser application.

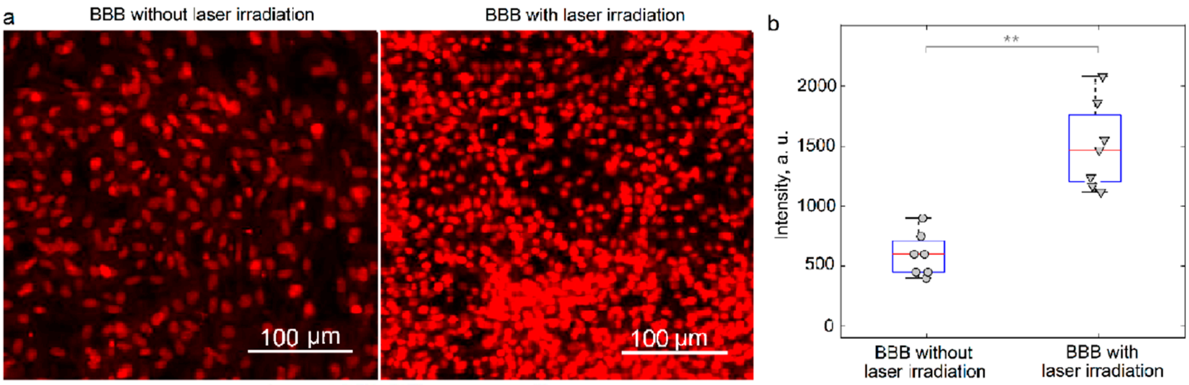

Figure S1. Analysis of the ROS production in the *in vitro* BBB model: a - The confocal imaging of DHE-IF in the groups, including the BBB cells without the 1268 nm laser irradiation and the BBB treated by the single 1268 nm laser irradiation (70 mV); b – The quantitative analysis of the ROS production in the tested groups. The data are presented as Means ± SEM, p<0.001 between groups, the Wilcoxon, Mann-Whitney U tests.

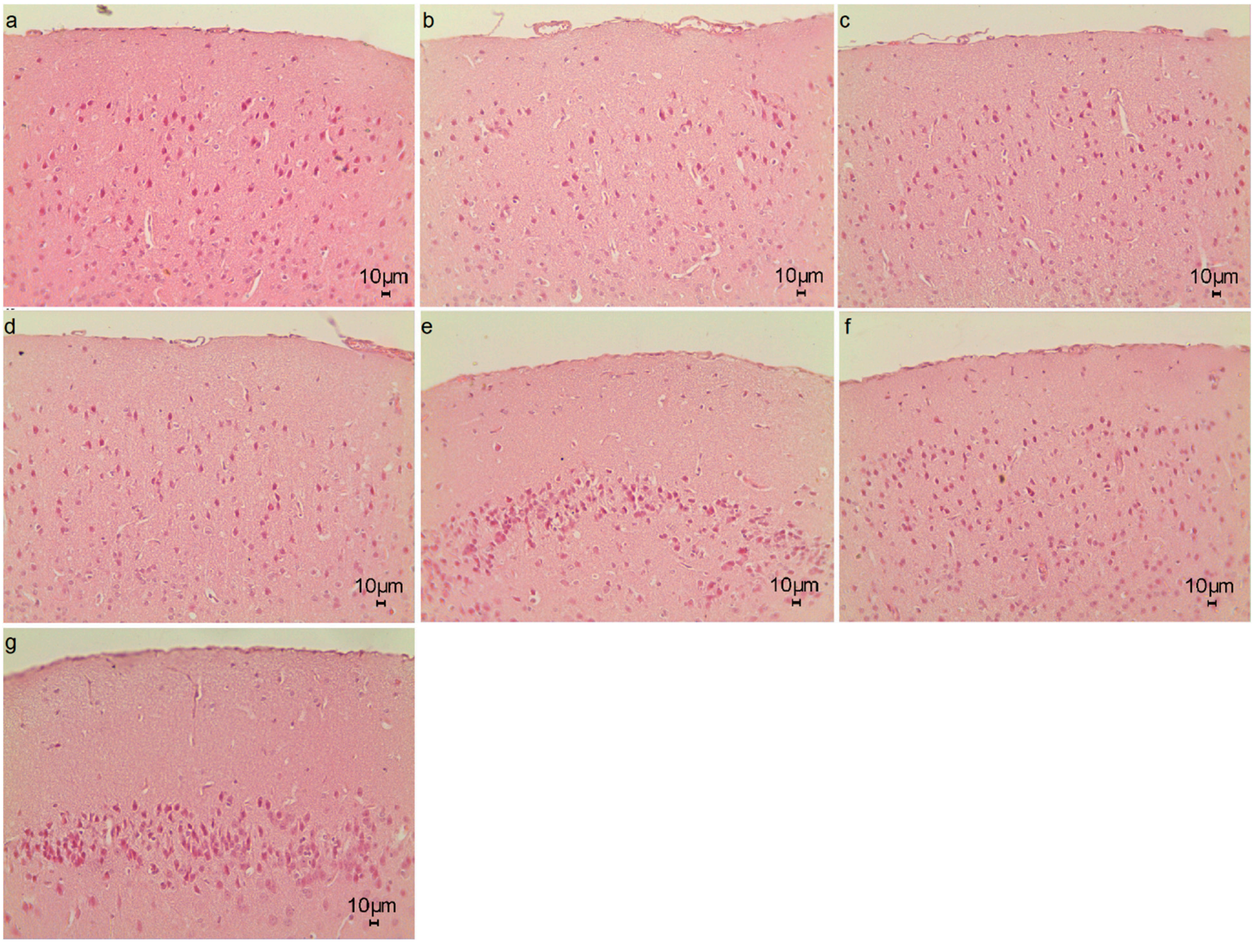

Figure S2. The histological analysis of the brain tissues without (a) and 1h (b-d, 70-80-90 mW, respectively) and 4hrs (e-g, 70-80-90 mW, respectively) after the laser 1268 nm irradiation, n=7 in each group

Table S2. Temperature (^{0}C) at the external surface of skull and the brain cortex without and after the 1268 nm laser irradiation in healthy mice

Intensity of the laser 1268 nm irradiation, mW	No Laser	10	20	30	40	50	60	70	80	90
The skull external surface	33.12±0.10	32.15±0.12	33.10±0.13	33.04±0.09	32.06±0.11	33.20±0.15	35.03±0.04	35.19±0.11	35.12±0.06	35.06±0.10
Under the skull on the cortex surface	37.11±0.12	37.22±0.09	37.08±0.14	37.00±0.03	37.26±0.10	37.22±0.31	37.86±0.25	37.94±0.18	37.88±0.21	37.76±0.02
